# Two Cases of Ectopic Gestation—Retention of the Fetus for Eight and Thirteen Years Respectively—Operation—Cure

**Published:** 1895-08

**Authors:** Matthew D. Mann

**Affiliations:** Buffalo, N. Y., Professor of gynecology, Medical Department, University of Buffalo.


### TWO CASES OF ECTOPIC GESTATION—RETENTION OF THE FETUS FOR EIGHT AND THIRTEEN YEARS RESPECTIVELY—OPERATION—CURE.

BY MATTHEW D. MANN, A. M., M. D., Buffalo, N. Y.,
Professor of gynecology, Medical Department, University of Buffalo.

IT IS generally supposed that when an ectopic pregnancy has reached full term and the child has died, the danger is, to a great extent, over; and, after a longer period, it is usually taken for granted that all danger is past. That this is a very great mistake, a study of the two cases which are here related will conclusively prove. Both cases go to show that, no matter how long a period may have elapsed after the death of the child, the woman is still in danger.

CASE I.—Mrs. C. B., age 33, and married twice; to her second husband eight years before coming under my care.

Soon after her first marriage she became pregnant. Everything seemed to be natural until the eighth month, when she was taken with all the symptoms of labor, but the child did not seem to advance. A physician tried to apply the forceps, but failed. The labor pains soon ceased, and the true condition having been recognised the patient refused all further interference.

She was afterward seen by Drs. Bartlett, J. P. White and Wyckoff, who all concurred in the diagnosis of extra-uterine pregnancy. The patient soon regained her ordinary health, the tumor diminished in size and gave her no further inconvenience. She was married five years afterward to her second husband, by whom she has had three children.

I was called to see her first in 1888, when I found her suffering from very considerable pain in the region of the gall-bladder. I made a diagnosis of probable gall-stones. At that time I also examined the abdomen and found what I supposed was a lithopedion. I remarked then that if it ever caused her any trouble she would do better to have it removed. She laughed and said that it had been there so long she did not think it would harm her any now.

On October 18, 1891, I was called to see her again in consultation with Dr. Bartlett. I found the patient suffering very much and very eager for relief. She stated that two months before she had noticed berry-seeds in the urine, and since that time had passed large quantities of what she called pus through the urethra. An inspection of the matter passed showed it to be the contents of the upper intestine. Nutrition had become considerably impaired from loss of food in this way. At times she felt very sharp pains in the back and the bladder was in a constant state of irritation. She stated she was willing to undergo anything and was clamorous for immediate relief.

I made the diagnosis of either an intestinal opening into the sac which contained the fetus and a second opening into the bladder, or else that an abscess had formed in one of the tubes which in turn had communicated with the intestine and bladder. The former seemed the more probable condition.

On October 22d, the patient having been removed to the General Hospital, I opened the abdomen. Before the operation one could feel a large hard mass in the lower portion of the abdomen, reaching nearly to the umbilicus. It was irregular in outline, with little projections here and there, feeling not unlike knees and elbows. The mass seemed very hard, as though it might have undergone calcareous degeneration. The intestines lay over the tumor, as could be made out by percussion. After the patient was anesthetised, it was found that the tumor was slightly movable.

On opening the abdomen with a long incision I came upon a sac with the omentum spread out over it and firmly attached. Getting my hand down to the side of the tumor, I managed to pass it between the upper part of the tumor and the omentum. Gathering this into a mass it was ligated in two places and cut between the ligatures. After the omentum was out of the way I found the tumor non-adherent, except that the intestines were attached to it in several places. The sac had now become quite movable and I could raise it from the pelvis.

Before going further I decided to open the sac and remove its contents. I did so, and came upon the foulest smelling mess I ever encountered. The first thing I touched was one of the bones of the fetal skull; after pulling this away the other cranial bones came into view, one by one. Then I came upon what was left of the fetus, a mass of decomposing flesh, bathed in pus, intestinal contents and urine. Some of the soft parts of the fetus had been preserved, and in places there were deposits of lime salts, but not to any great extent—certainly not to the extent of making the fetus a lithopedion. The heart, surrounded by the pericardium, the lungs, diaphragm, liver, portions of the intestine and bladder were all distinctly recognisable, although the extremities were completely decomposed, only the bones being left. After removing the greater part of the fetus I found long diverticula in the sac, some corresponding to the long bones in the arm and others to those of the leg.

After removing the sac from the pelvis and cleaning it out, I packed it with dry absorbent cotton to prevent the escape of any of its contents into the abdominal cavity. From the very first, wet towels and sponges had been packed around it, so that the abdominal cavity remained perfectly clean.

The next step was to separate the intestines. Five or six loops were adherent, and in two of them—one of the small and the other of the large intestine—I found openings connecting with the sac, through which I could readily pass two or three fingers. The portions around these openings were carefully disinfected with the peroxide of hydrogen, and packed in towels and placed on one side. After all the intestines had been separated, the sac was cut away, after carefully securing the pedicle. A portion of the sac was adherent to the bladder, and at this point there was an opening into this viscus which would easily admit of my finger. Evidently fecal matter had passed from the intestine into the sac, and then out through the bladder.

On carefully examining the intestines, I found the openings were both so large that to sew them up would cause great danger of stricture. Dr. Roswell Park suggested that the openings be sewed together so as to form an intestinal anastomosis. To this I objected, as it seemed to me that there would be great danger of starving the patient to death if the small intestine, at so high a point, was made to empty into the lower portion of the large intestine. I, therefore, decided at once to resect both intestines. In order to save time I asked Dr. Park, who was present as a spectator, to undertake one of the resections. This he kindly did, taking the large intestine, which was upon the side opposite to me. In this way at least twenty minutes of time was saved, which time, if the operation had been extended, might have cost the patient her life. The intestines were united with silk by the Lembert suture, slightly modified. Instead of an interrupted suture, four or five uninterrupted stitches were taken and then the ends tied; then, again, four or five more uninterrupted sutures, and so on around. The portion of mesentery opposite to the resected intestine was tied with catgut. After the intestines had been properly united by two rows of sutures, the hole in the bladder was sewed up with an uninterrupted peritoneal suture. As the abdomen seemed to be perfectly clean it was not washed out, nor was a drainage-tube introduced.

The patient suffered a good deal from shock, but soon rallied, and did perfectly well until the eighth day. She then complained of pain in the neighborhood of the liver; in the evening she had a chill; temperature, 104°; pulse, 135. Next morning I found her, still with rapid pulse and high temperature, complaining of great pain and tenderness in the neighborhood of the gall-bladder. A distinct tumor could be recognised at this point. I administered frequent small doses of calomel and applied poultices over the swelling. This soon subsided, pulse and temperature came down, and the patient made an otherwise uninterrupted recovery.

For the first two days she had absolutely no food, only a very little water and hot tea. The catheter was passed at first at frequent intervals. There was no blood in the urine at any time. On the fourth day the nausea and vomiting ceased, having been persistent up to this time. She then began to take small quantities of hot tea, wine and chicken broth. On the third day the bowels moved slightly after an enema. On the fourth day they moved five times without any cathartic. The patient left the hospital on the twenty-eighth day, apparently perfectly well.

A more detailed description of the tumor in this case may be of interest. The sac was on the left of the uterus. On the left side of the sac I found a normal Fallopian tube and ovary. On the right side of the uterus there was also a tube and ovary. The walls of the sac seemed to resemble very much those of a distended uterus; and what might be called the pedicle of the tumor was attached to the uterus in the neighborhood of the isthmus and seemed to merge directly into it. From these facts it seemed to me quite evident that this was not strictly an extra-uterine pregnancy, but rather a pregnancy in one horn of a double uterus.

The method of treating the pedicle should also be more carefully described. I first secured it with an elastic ligature, and on removing this I found there was little or no hemorrhage. I, therefore, made a wedge-shaped excision, brought the edges together and turned them in, and closed the whole with the cobbler's stitch suture, sewing the pedicle through and through with catgut. There was no oozing or leakage from it at all and the result seemed to justify the method.

This case certainly presents many features of interest. I have, in a previous paper, alluded to the case as one of lithopedion, and have used it as an argument to show the innocuousness of a retained fetus. I shall have to take this all back. The intestinal complication rendered the case very much more difficult to manage than it would have been otherwise. Had it not been for this, and the adhesion to the bladder, the sac could have been removed as easily as any simple ovarian cyst. A few months later two calculi formed in the bladder. They were removed by my associate, Dr. Crockett.

CASE II.—In January, 1892, Dr. W. W. Smith, of Avoca, N. Y., brought to me Mrs. S., *æt.* 39, and gave the following history of her case:

He stated that she had been married for twenty years, and that two years after her marriage she bore a living child. At that time she was very ill for three or four weeks with some septic trouble, and after it many adhesions remained in the pelvis around the uterus. She gradually regained her health, and twelve years later, eight years before coming to me, became pregnant again. At the end of the first month of pregnancy she had severe pain in the abdomen, followed by some shock. During the first three months of her pregnancy she had six or eight of these attacks. Each time there would be a slight flow from the uterus. At the end of the ninth month labor set in, but there was no dilatation of the uterus. There was tumultuous action of the child, and great suffering on the part of the woman; but, finally, all the symptoms subsided, menstruation returned, and she regained her usual health.

Three weeks before coming to me, after a sudden exertion, she was taken with violent pain in the abdomen with slight shock, and the abdomen became very tender. Dr. M. A. Crockett saw her that evening. He found her general condition good, the tenderness over the abdomen much diminished, and no change in the form or position of the tumor. Following this, there were several attacks of pain with tenderness of the abdomen, but not so severe as at the time of the first attack.

When brought to me she had not menstruated for ten weeks and believed herself pregnant. At that time I found her rather pale, with an anxious expression and a strong pulse. Thinking that she might have become pregnant the second time, I etherized her, dilated the cervix, and thoroughly explored the uterine cavity. Nothing could be removed by the curette or by the forceps, and no abortion followed. At the time she was under ether a careful examination of the abdomen was made, and I found an enlargement, most marked on the right side, quite high. The tumor seemed to be firm, resisting and quite irregular in outline, about the size of a uterus at the sixth month. There was an area of flatness over the tumor and no evidences of fluctuation. The uterus seemed to be high and pushed very much over to the left side. Diagnosis of extra-uterine pregnancy with retained fetus was made; but what was the cause of the recent symptoms we were unable to decide.

It was determined to make an exploratory celiotomy, which was done on January 27, 1892, assisted by Dr. Crockett, and in the presence of several gentlemen. An incision about six inches long was made, in order to have plenty of room to work. Adhesions were found to be very numerous. The omentum and intestines were united to the sac, and several inches of the small intestine had to be carefully dissected away. There was not a great deal of hemorrhage, but considerable oozing from the separated adhesions. After the omentum and intestines had been separated, the sac was opened by a median incision and a full-term fetus extracted. There was no odor, and the child showed no signs of decomposition. The placenta occupied the bottom of the sac, extending up along the lower wall to the site of the incision. It was firmly adherent, but not very vascular. It was left *in situ*, as it was found almost impossible to separate it. The fetus was a female, somewhat shrunken and mummified. At several points there were areas of denuded skin, looking as though the parts had been adherent to the sac and separated at the time of its removal.

The patient made a slow recovery; the wound suppurated and was packed and dressed for a long time. The placenta finally came away piecemeal, and it was not until the seventh week that the evening temperature remained below 100°. The patient left the hospital with a sinus, but this gradually closed, and she ultimately entirely recovered her health.

What was the cause of the outbreak a short time before coming to the hospital was never determined, as the result of the operation showed nothing to account for it. This, like the case already related, shows that a fetus within the abdomen of the mother, although it may remain quiet for years and cause no trouble, will almost certainly in the end do harm. This patient did not retain the fetus as long as the preceding one; but, had it not been removed, it is altogether likely that suppuration and decomposition would have eventually occurred.

The certain lesson which these cases teach is that a fetus outside of the uterus or within an undeveloped horn of a double uterus is a constant menace and source of danger to the mother, and, no matter how well she may seem at the time, nor how little indication there may be for operation, nor how long it may have been retained, it is wiser to advise the immediate removal of such a fetus whenever it may be discovered.

This, of course, does not involve the question of operation before the death of the child. That is entirely outside of the points suggested by these cases. But, after the child is once dead, there can be no use in waiting for any great length of time. A few weeks to allow of the stoppage of the circulation in the placenta has been advised. Waiting for this to occur caused me to lose one case which, had I operated on a few days sooner, I doubtless could have saved. This case has already been published. A dead fetus within the abdomen of the mother, although it may be producing no symptoms, may be very aptly likened to a charge of dynamite: it takes only very little to make it go off, and then the damage done is apt to be serious.

37 Allen Street.

